# The Accuracy of Diagnostic Methods for Diabetic Retinopathy: A Systematic Review and Meta-Analysis

**DOI:** 10.1371/journal.pone.0154411

**Published:** 2016-04-28

**Authors:** Vicente Martínez-Vizcaíno, Iván Cavero-Redondo, Celia Álvarez-Bueno, Fernando Rodríguez-Artalejo

**Affiliations:** 1 Universidad de Castilla-La Mancha, Health and Social Research Center, Cuenca, Spain; 2 Universidad Autónoma de Chile, Facultad de Ciencias de la Salud, Talca, Chile; 3 Universidad Autónoma de Madrid, Preventive Medicine and Public Health, Madrid, Spain; Florida International University Herbert Wertheim College of Medicine, UNITED STATES

## Abstract

**Objective:**

The objective of this study was to evaluate the accuracy of the recommended glycemic measures for diagnosing diabetic retinopathy.

**Methods:**

We systematically searched MEDLINE, EMBASE, the Cochrane Library, and the Web of Science databases from inception to July 2015 for observational studies comparing the diagnostic accuracy of glycated hemoglobin (HbA1c), fasting plasma glucose (FPG), and 2-hour plasma glucose (2h-PG). Random effects models for the diagnostic odds ratio (dOR) value computed by Moses’ constant for a linear model and 95% CIs were used to calculate the accuracy of the test. Hierarchical summary receiver operating characteristic curves (HSROC) were used to summarize the overall test performance.

**Results:**

Eleven published studies were included in the meta-analysis. The pooled dOR values for the diagnosis of retinopathy were 16.32 (95% CI 13.86–19.22) for HbA1c and 4.87 (95% CI 4.39–5.40) for FPG. The area under the HSROC was 0.837 (95% CI 0.781–0.892) for HbA1c and 0.735 (95% CI 0.657–0.813) for FPG. The 95% confidence region for the point that summarizes the overall test performance of the included studies occurs where the cut-offs ranged from 6.1% (43.2 mmol/mol) to 7.8% (61.7 mmol/mol) for HbA1c and from 7.8 to 9.3 mmol/L for FPG. In the four studies that provided information regarding 2h-PG, the pooled accuracy estimates for HbA1c were similar to those of 2h-PG; the overall performance for HbA1c was superior to that for FPG.

**Conclusions:**

The three recommended tests for the diagnosis of type 2 diabetes in nonpregnant adults showed sufficient accuracy for their use in clinical settings, although the overall accuracy for the diagnosis of retinopathy was similar for HbA1c and 2h-PG, which were both more accurate than for FPG. Due to the variability and inconveniences of the glucose level-based methods, HbA1c appears to be the most appropriate method for the diagnosis diabetic retinopathy.

## Introduction

In 1997, the Expert Committee on the Diagnosis and Classification of Diabetes Mellitus stated that the diagnosis of diabetes should focus simultaneously on plasma glucose concentrations and its long-term microvascular complications, particularly diabetic retinopathy [1]. In 2009, a report from the International Expert Committee (IEC) proposed glycated hemoglobin (HbA1c) as an appropriate test for diagnosing diabetes [2]. The American Diabetes Federation [3] and the World Health Organization [4] reinforced this recommendation and maintained that both fasting plasma glucose (FPG) and 2-hour plasma glucose (2h-PG) after a 75-g oral glucose tolerance test (OGTT) are appropriate tests for the diagnosis of diabetes in non-pregnant adults.

The variety of biomarkers for diagnosing diabetes poses a challenge for clinicians and health planners [5]. Clinicians should consider the advantages and disadvantages of using the biomarkers and decide which test, or which combination of tests in a pre-specified order, should be used for each type of patient [6]. The advantages of HbA1c are it is not modified by acute events, such as stress or vigorous physical exercise, and that it has greater pre-analytical stability and renders more reliable results than glucose-based tests. However, it has also been reported that HbA1c levels substantially depend on various non-glycemic factors, such as iron or vitamin B12 deficiency, renal failure, or variables related to the lifespan of red blood cells [7]. Moreover, neither the FPG nor the 2h-PG tests are influenced by individual susceptibility to the glycation of hemoglobin, genetic factors and individual characteristics [8], such as age or ethnicity. Furthermore, the costs of determining HbA1c are higher than those of FPG.

Diabetic retinopathy is an early diabetes-related complication that is a good criterion for comparing the diagnostic accuracy of diabetes biomarkers [1]. The DETECT-2 project, an international pool of nine studies from five countries, recently re-examined the relationship between glycemic measures and retinopathy. It was suggested that the current diabetes diagnostic level for FPG could be lowered from 7.0 to 6.5 mmol/L and that an HbA1c level of 6.5% (47.5 mmol/mol) is a suitable alternative diagnostic criterion [9]. The World Health Organization, based on the level above which the risk of developing micro- and macrovascular complications increases, has also recommended the use of 6.1 mmol/L as FPG cutoff point for the diagnosis of impaired fasting glucose; furthermore, the ADA recommended lowering this threshold from 6.1mmol/l to 5.6mmol/l [3, 4]. However, to our knowledge, no previous study has comprehensively reviewed and compared the accuracy of the main glycemic measures to identify diabetes-specific retinopathy.

Thus, we conducted a systematic review and meta-analysis of the literature to evaluate the accuracy of HbA1c, FPG and 2h-PG for diagnosing diabetic retinopathy.

## Methods

### Literature search

A literature search was conducted in MEDLINE (via PubMed), EMBASE, the Cochrane Central Register of Controlled Trials, the Cochrane Database of Systematic Reviews and the Web of Science databases from their inception to July 17, 2015. Three comprehensive search themes were combined using Boolean operators: [“HbA1c” OR “glycated hemoglobin” OR “glycated hemoglobin” OR “hemoglobin A1c” OR “glucose” OR “fasting glucose”] AND [“threshold” OR “cut-off” OR “cut point” OR “sensitivity” OR “specificity” OR “diagnostic” OR “differential diagnosis”] AND [“microvascular complications” OR “retinopathy” OR “retinal”]. The reference lists of the retrieved articles were reviewed for additional studies. The literature search was performed independently by two reviewers (IC and CA), and inconsistencies were resolved via conference.

### Selection criteria

We aimed to identify original articles analyzing the HbA1c, FPG and 2h-PG thresholds associated with an increased frequency of retinopathy. The following inclusion criteria were used: i) study participants were individuals aged ≥18 years; ii) index tests used were HbA1c, FPG and 2h-PG; iii) an outcome of diabetic retinopathy at any stage; and iv) study designs including cross-sectional, case-control, or cohort studies, with either prospective or retrospective data collection. The exclusion criteria were as follows: i) insufficient data to calculate sensitivity or specificity; ii) studies conducted only with diagnosed diabetic individuals; iii) studies conducted on gestational diabetes; and iv) studies written in a language other than English or Spanish. When multiple articles reported data from the same study, the most recent article was selected.

### Data extraction and quality assessment

The following data were collected from each study were included in this review: 1) author identification, 2) year of publication, 3) country of the study, 4) year of data collection, 5) ophthalmic examination test, 6) age of the participants, 7) number of participants, 8) prevalence of retinopathy and 9) parameters summarizing the accuracy of the test (cut-off, sensitivity, specificity, area under curve (AUC) and the diagnostic odds ratio (dOR)).

We used the Quality Assessment of Diagnostic Accuracy Studies-2 (QUADAS-2) tool to evaluate four domains of each study: patient selection, index test, reference standard and flow of patients and timing of the tests. Each domain was evaluated in terms of the risk of bias, and the first 3 domains were also evaluated in terms of concerns regarding the applicability of the results [10].

Data extraction and quality assessment were independently performed by IC and CA, and inconsistencies were managed by consensus.

### Statistical analysis and data synthesis

This study was reported according to the PRISMA [11] statement (Table A and Figure A in S1 File) and the recommendations of the Cochrane Collaboration Handbook [12]. The sensitivity, specificity, AUC and dOR as well as their corresponding 95% confidence intervals (CIs) were calculated for HbA1c, FPG and 2h-PG in each included study. Although the protocol of this meta-analysis specified that at least five studies were required in a subgroup to conduct the pooled estimations, a meta-analysis including only four studies is provided at (Table B in S1 File).

Hierarchical summary receiver operating characteristic curves (HSROC) were used to summarize the overall test performance. The HSROC have been proposed to estimate the performance of diagnostic tests on data from a meta-analysis, and the AUC is not only useful to evaluate not only the curve but also the strength of the heterogeneity [13]. To reach a threshold of excellent accuracy, the AUC must be in the region of 0.97 or higher. An AUC of 0.93 to 0.96 is very good and an AUC of 0.75 to 0.92 is good. An AUC less than 0.75 may be reasonable, but the test has evident shortcomings in its diagnostic accuracy [14]. When a study did not provide information about the AUC, it was calculated.

The dOR was computed using Moses’ constant of a linear model, which indicates that this approach relies on the linear regression of the logarithm of the dOR of a study (dependent variable) and on an expression of the positivity threshold of that study (independent variable). The dOR is a measure of the accuracy of the test data that combines sensitivity and specificity into a single value. The dOR values range from 0 to infinity, with higher values indicating a better discriminatory test performance (higher accuracy). A dOR of 1.0 indicates that a test does not discriminate between patients with the disorder and those without it [15].

Forest plots were used to display the sensitivity, specificity, AUC and dOR for each glycemic parameter in the reviewed studies. The heterogeneity of the results across studies was evaluated using the I^2^ statistical parameter. I^2^ values of <25%, 25–50% and >50% usually correspond to small, medium and large heterogeneity, respectively [16]. Given that in most cases the heterogeneity was large, the results of the different studies were pooled using a random-effects model with the Der Simonian and Laird method.

The separate influence of each study in the pooled dOR was estimated by recalculating the pooled estimate after the exclusion of individual studies. Finally, publication bias was visually evaluated using a funnel plot, as well as with the method proposed by Deeks [17].

Statistical analyses were performed using StataSE software, version 13 (StataCorp).

## Results

### Baseline Characteristics

**Fig 1:**
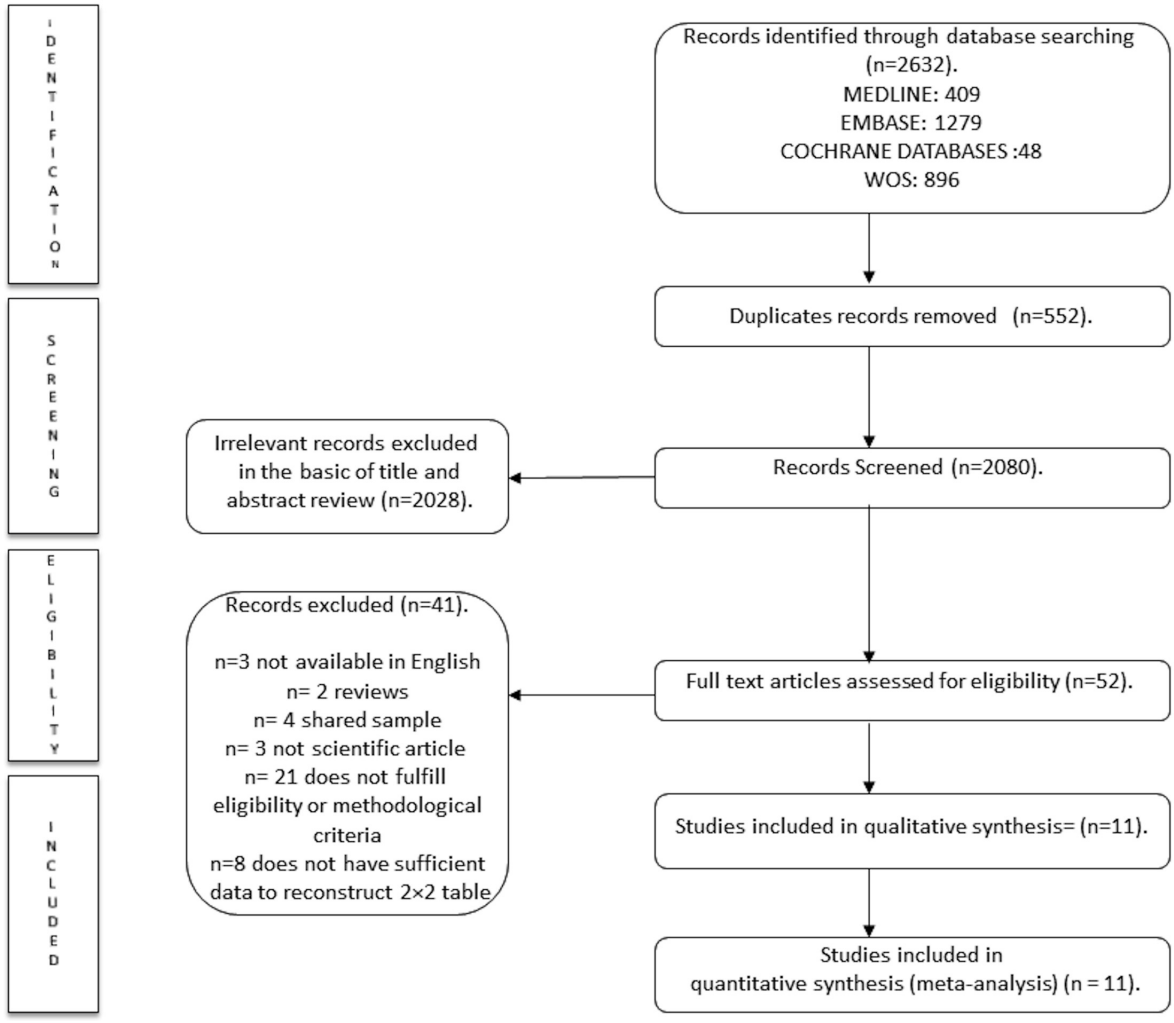
Literature search PRISMA consort diagram.

A total of 2,632 articles were retrieved from the literature search. After removing 552 duplicated articles, the titles and abstracts of 2,080 studies were screened. We excluded 2,028 studies that clearly did not fulfil all of the inclusion criteria or met at least one of the exclusion criteria, leaving 52 studies that were reviewed in full. Next, 41 of the studies were excluded following the full text reading (see study exclusion in References A in S1 File), and the remaining 11 articles were used for the final analysis (Fig 1) [18–28].

The 11 studies comprising this review included 45,686 participants. The studies were conducted in China, North America, Japan, Korea, India, Malaysia, France and Australia; one study was conducted among Pima Indians. The age of the participants ranged from 18 to 79 years. The retinopathy prevalence varied from 1.6% to 15.8% across the studies. All of the studies provided information on the global diabetic retinopathy prevalence, except one study that reported only moderate non-proliferative retinopathy [19]. All of the studies except for one, which also showed prospective data [28], had cross-sectional designs. Only four studies provided information regarding 2h-PG [19, 22, 27, 28]. Finally, one study provided several cut-offs for FPG; however, we selected the internationally recommended cut-off of 7.0 mmol/L for this analysis (Table 1).

**Table 1:** Characteristics of studies included in the meta-analysis. Sens: sensitivity; Spec: specificity; AUC, area under the curve; dOR, diagnostic odds ratio; FPG, fasting plasma glucose; 2h-PG, 2 hours plasma glucose.

								Diabetes retinopathy diagnosis				
Reference	Country	Study/Year data collection	Ophthalmic examination test	Age	n	Prevalence of retinopathy	Diagnostic test	Cut-off point	Sens (%)	Spec (%)	AUC	dOR
Sabanayagam et al. 2015^20^	India	SINDI/2007–09 SiMES/2004–06; SCES/2009–11	two 45° retinal images	56.4 (10.3) 57.0 (11.5) 54.6 (11.7)	3,740 3,596 5,834	4.4 3.8 1.6	HbA1c^a^ HbA1c^b^ HbA1c^c^	6.5 6.5 6.5	86.0 85.3 75.8	71.9 76.3 89.7	0.851 0.853 0.861	15.34 18.63 27.28
Mukai et al. 2014^21^	Japan	HISAYAMA study/2007–08	45° fundus photographs	49–70	2,681	1.9	HbA1c FPG 2h-PG	6.1 6.5 11.5	86.5 82.7 90.4	88.8 86.6 89.3	0.919 0.908 0.947	50.80 30.89 78.60
Park et al. 2014^22^	Korean	5th KNHANES/2011	45° nonmydriatic digital retinal image	>19 (44.3±0.4)	5,212	1.6	HbA1c FPG	6.2 6.3	93.9 82.6	89.7 91.2	0.953 0.908	134.06 49.20
Cho et al. 2013^23^	Korean	ANSUNG Cohort study/2009–10	45° nonmydriatic fundus photography	40–60 (63.3 ±8.6)	3,403	1.9	HbA1c FPG	6.6 6.0	76.2 69.8	84.2 77.1	0.830 0.730	17.06 7.78
Xin et al. 2012^24^	China	Health Examination Survey in Beijing/2010–11	45° colour digital images	18–79	2,551	2.9	HbA1c FPG 2h-PG	6.8 7.8 15.0	85.1 75.7 74.3	88.0 87.9 90.6	0.864 0.854 0.869	41.88 22.63 27.90
Massin et al. 2011^25^	France	DESIR/1994–96	Three nonmydriatic digital retinal photograph	30–65 (52)	700	3.2	HbA1c FPG	6.0 6.0	19.0 27.0	92.0 88.0	0.640 0.640	2.70 2.71
Jonas et al. 2010^26^	China	BEIJING eye study/2006	45° nonstereoscopic photograph of central fundus	>45 (60.4±10.0)	2,916	12.2	FPG	7.0	18.8	94.3	0.610	3.83
Cheng et al. 2009^27^	USA	NHANES/2005–06	Two 45° nonmydriatic colour digital retina image	>40 (56)	1,066	11.0	HbA1c FPG	5.5 5.8	80.0 58.0	37.0 64.0	0.710 0.650	2.35 2.45
Wong et al. 2008^28^	Australia, USA	BMES/1997–99; AusDiab/1999–2000; MESA/2002–04	six 30° retinal photographs two 45° retinal photographs	>49 >25 45–84	3,162 2,182 6,079	11.5 9.6 15.8	FPG^a^ FPG^b^ FPG^c^	7.0 7.0 7.0	14.8 39.0 24.4	95.8 80.8 91.4	0.560 0.610 0.600	3.96 2.70 3.42
Miyazaki et al. 2004^29^	Japan	HISAYAMA study/1998	45° fundus photographs	40–79	1,637	2.3	HbA1c FPG 2h-PG	5.7 6.4 11.1	86.5 86.5 86.5	90.1 87.3 89.6	0.945 0.900 0.960	58.30 44.04 78.90
McCance et al. 1994^30^	USA (Pima Indian)	Gila River Indian Community/1982–90	Direct ophthalmoscopy examination	>25	927	3.23	HbA1c FPG 2h-PG	7.8 9.3 12.6	65.6 68.8 87.5	87.6 87.7 80.2	0.821 0.822 0.841	13.47 15.72 28.35

^a^,^b^ and ^c^ indicate different subgroups of participants in that study.

### Study Quality

As evaluated with QUADAS-2, all of the studies included information regarding the seven quality items. However, the studies had shortcomings in two domains: the index test and the reasons for excluding participants. In fact, most studies interpreted their results without reference to a standard (HbA1c: 78%; FPG: 60%; and 2h-PG: 75%) and only considered the pre-specified index test threshold (Table C and Figure A in S1 File).

### Meta-analysis

**Fig 2:**
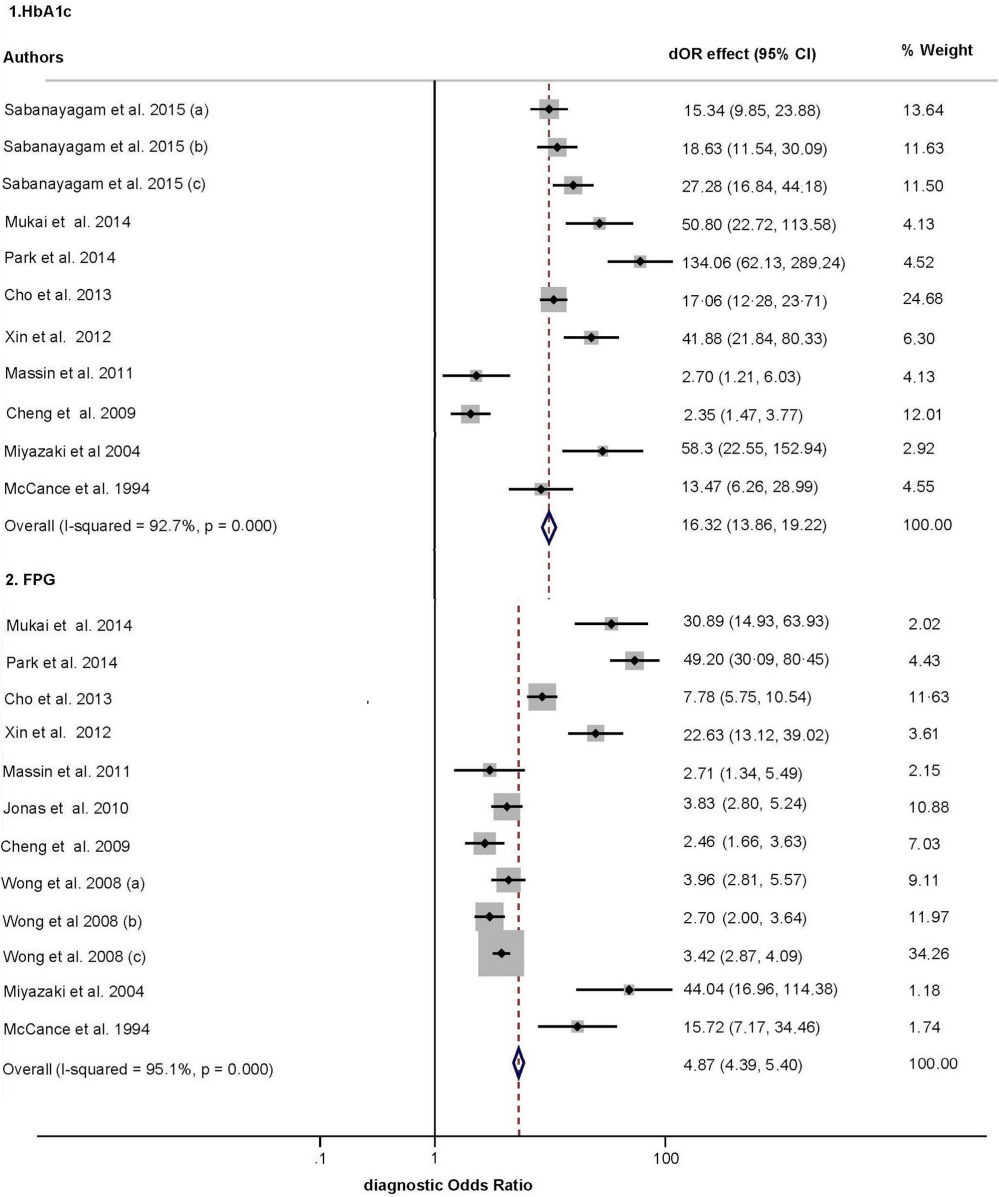
Forest plot of the diagnostic odds ratio (dOR) of each index test in the reviewed studies. CI: confidence interval; (a), (b) and (c) indicate different subgroups of participants in that study, as defined by setting (Table 1).

Fig 2 depicts the dOR funnel plots of HbA1c and FPG. There was substantial heterogeneity across the studies in the dOR of retinopathy based on HbA1c (I^2^ = 92.7%) and FPG (I^2^ = 95.1%). The pooled dOR for the diagnosis of diabetic retinopathy was 16.32 (95% CI, 13.86–19.22; p < 0.001) for HbA1c and 4.87 (95% CI, 4.39–5.40; p < 0.001) for FPG. The pooled sensitivity, specificity, positive likelihood ratio (PLR), negative likelihood ratio (NLR), dOR and AUC for HbA1c and FPG are shown in Table 2 (Figure B, C, D and E in S1 File depict sensitivity, specificity, PLR and NLR funnel plots, respectively)

**Table 2:** Pooled accuracy parameters in the diagnosis of diabetic retinopathy, by index test. Values in parentheses are 95 per cent confidence intervals. FPG: fasting plasma glucose, PLR: positive likelihood ratio, NLR: negative likelihood ratio, dOR: diagnostic odds ratio, AUC: area under receiver operating characteristic curve.

	N°. of studies	Sensitivity (%)	Specificity (%)	PLR	NLR	dOR	AUC
HbA1c	11	82.0 (76.0–87.0)	84.0 (83.0–85.0)	5.29 (2.56–10.91)	0.21 (0.10–0.44)	16.32 (13.86–19.22)	0.837 (0.781–0.892)
FPG	12	42.5 (39.8–45.3)	88.2 (87.2–89.3)	4.57 (2.04–10.24)	0.40 (0.20–0.82)	4.86 (4.39–5.40)	0.735 (0.657–0.813)

**Fig 3:**
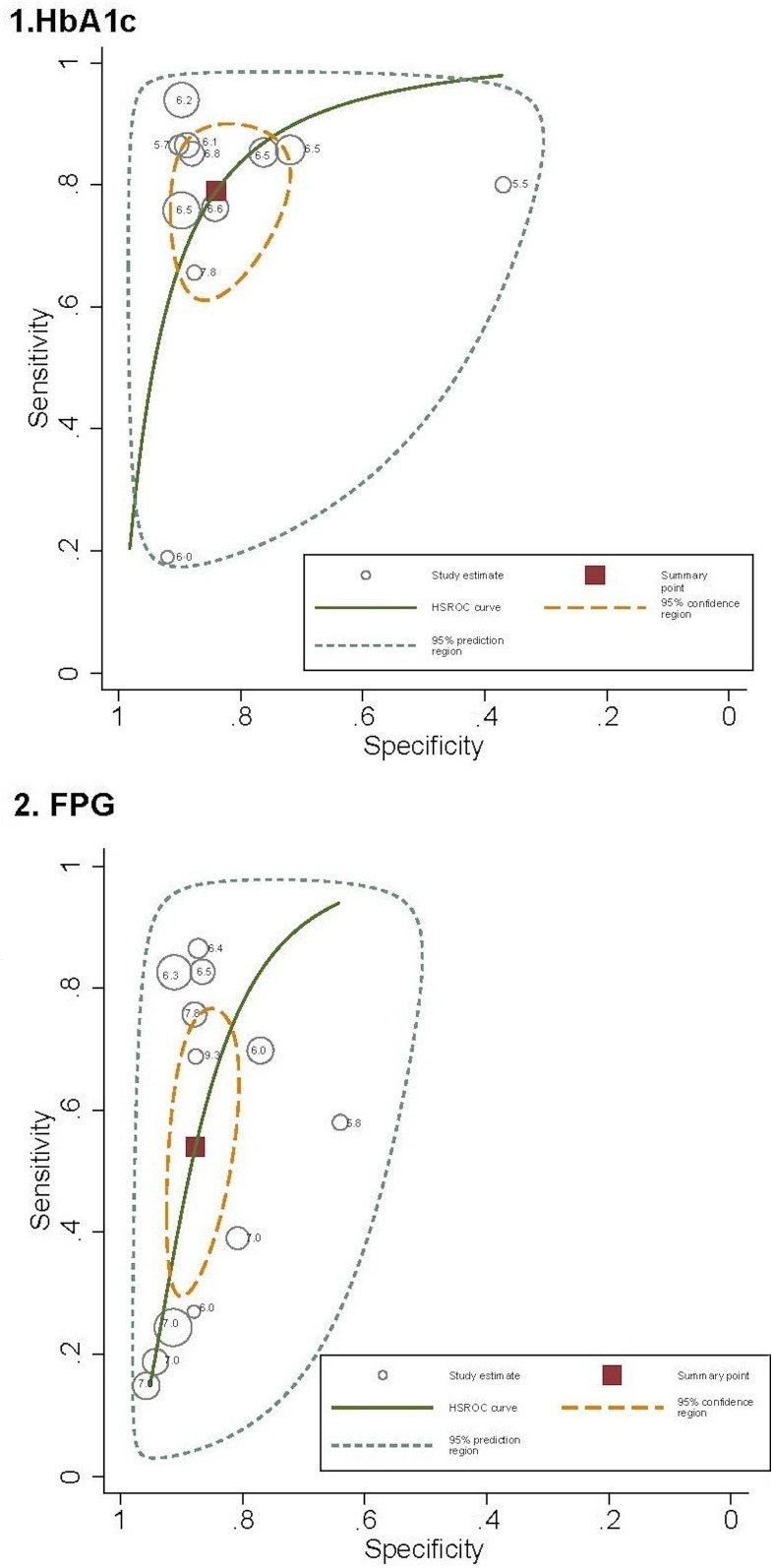
Hierarchical summary receiver operating characteristic (HSROC) curves summarizing the ability of glycated haemoglobin (HbA1c) and fasting plasma glucose (FPG) to identify diabetes retinopathy.

The area under the HSROC (Fig 3) estimating the discriminating accuracy of HbA1c for identifying retinopathy was 0.837 (95% CI: 0.781–0.892; p < 0.001) and was 0.735 (95% CI: 0.657–0.813; p < 0.001) for FPG. The 95% confidence region for the point that summarized the overall test performance included studies in which the test cut-offs ranged from 6.1% (43.2 mmol/mol) to 7.8% (61.7 mmol/mol) for HbA1c and from 7.8 to 9.3 mmol/L for FPG.

When we estimated the pooled accuracy parameters from the four studies that evaluated the diagnostic performance of HbA1c, FPG and 2h-PG in the same sample, the pooled dOR was 34.68 (95% CI, 23.56–51.03; p < 0.001) for HbA1c, 24.79 (95% CI, 17.40–35.32; p < 0.001) for FPG and 32.39 (95% CI, 25.27–41.51; p < 0.001) for 2h-PG. In addition, the pooled AUC was 0.882 (95% CI: 0.835–0.930; p < 0.001) for HbA1c, 0.868 (95% CI: 0.824–0.912; p < 0.001) for FPG and 0.916 (95% CI: 0.870–0.963; p < 0.001) for 2h-PG (Table C in S1 File).

### Sensitivity analysis for the effect of individual studies

When the impact of individual studies was examined by removing studies from the analysis one at a time we observed that the pooled dOR estimation for HbA1c increases after removing data from the Cheng et al. [25] study (dOR, 21.26 [95% CI: 17.86–25.31]). The pooled dOR for FPG also increases after removing data from the Wong et al. [26] study (dOR, 5.4 [95% CI: 5.14–6.64]), but decreases after removing data from the Park et al. [27] study (dOR, 4.37 [95% CI: 3.93–4.86]) (Figure F in S1 File).

### Publication bias

The asymmetry test, using Deek’s method [17], did not suggest the existence of publication bias either for HbA1c (intercept, 2.85 [95% CI: −0.65–5.76]; p = 0.054) or for FPG (intercept, 0.67 [95% CI: −0.29–1.63]; p = 0.151) (Figure G in S1 File).

## Discussion

The most recent recommendations propose HbA1c as a good test for diagnosing diabetes in non-pregnant adults and also include FPG and 2h-PG as appropriate methods [3, 4]. Thus, which of the recommended tests should be used remains controversial. In our meta-analysis of 11 studies, HbA1c performed better than FPG in identifying individuals with diabetic retinopathy. Moreover, our data indicate that the three glycemic tests have sufficient diagnostic accuracy on diabetic retinopathy in clinical practice, supporting the current international recommendations.

Our meta-analysis of the four studies [19, 22, 27, 28] that compared these three tests in the same set of patients showed that, overall, 2h-PG and HbA1c have similar accuracy estimates for diabetes retinopathy in terms of the dOR and AUC and are better than FPG. In recent decades, the 2h-PG after a 75-g oral glucose tolerance test (OGTT) has been the preferred test for confirming a diagnosis of diabetes in clinical practice, but because it is time-consuming and labor-intensive [29], both the FPG and HbA1c tests are considered good alternatives [2, 4].

Although the pooled specificity in the meta-analysis of the 11 studies comparing HbA1c and FPG was similar, the pooled sensitivity for HbA1c was 2-fold higher than that for FPG, and the pooled dOR was almost 4-fold higher. Regarding the low sensitivity of FPG, the Diabetes Prevention Program [30] and NHANES [25] reported that 8% of individuals with a FPG below diabetic thresholds had retinopathy. Thus, using the recommended FPG cut-off of 7.0 mmol/L for the diagnosis of diabetes [2, 3, 4], a not negligible percentage of cases of diabetic retinopathy would be undiagnosed. Other advantages of HbA1c are that it can be measured in a non-fasting state and it has good pre-analytical stability and low day-to-day variability. However, HbA1c has some limitations: diabetes is defined by high blood glucose rather than by glycation of proteins and HbA1c does not reflect postprandial glycaemia [5].

Authors have questioned the use of diabetes retinopathy as the gold standard for the diagnosis of diabetes because no uniform glycemic threshold for the presence of retinopathy has been found across populations [26]. Moreover, most studies relating HbA1c to retinopathy have been cross-sectional and have not excluded individuals with diagnosed diabetes (even if treated with hypoglycemic drugs, and the reported thresholds were dependent on the statistical methods used, the definition of retinopathy, and factors influencing HbA1c levels, such as individual susceptibility to glycation and aging. However, currently, no other clinical diagnostic standard exists for diabetes.

Meta-analyses of diagnostic tests synthetize the performance of a test providing a pooled estimation of diagnostic accuracy parameters, and also estimates a summary point (a summary sensitivity and specificity estimates) and a HSROC, but not allows the identification of the optimal cut-off point [31]. However, the cut-offs within the 95% confidence region for HbA1c ranged from 6.1% (43.2 mmol/mol) to 7.8% (61.7 mmol/mol) and from 7.8 to 9.3 mmol/L for FPG. These findings support the cut-offs proposed by the International Expert Committee for the diagnosis of diabetes using HbA1C, but not for FPG [2].

As is common in diagnostic meta-analyses, all of the estimations of the diagnostic accuracy were performed considering the large variability across individual studies. A substantial part of this variability is derived from a threshold effect due to the differences in the thresholds used to determine positivity in the tests. Factors influencing the threshold effect across the studies include the criteria for the diagnosis of retinopathy, the statistical methods used for defining cut-offs, and the assay methods used to measure diagnostic tests, particularly HbA1c. The wide clinical spectrum of patients included in the studies is also responsible for a substantial proportion of variability across the studies. While participants in some studies are a representative sample of the general population, other studies included selected samples with a known high prevalence of diabetes. Moreover, some studies removed individuals undergoing antidiabetic drug treatment from the analyses, and others accounted for potential modifiers, such as age or hypertension. In fact, the threshold effect and the wide spectrum of patients could explain the “shoulder arm” found in the HSROC graphics, which partially results from the inverse correlation between the sensitivity and specificity. Note that this correlation and the large variability in diagnostic accuracy across the studies support the use of HSROC because they explicitly addresses the relationship between sensitivity and specificity using the threshold [32] and account for inter-study heterogeneity.

In the sensitivity analysis we observed that the estimate of the pooled dOR decreases after removing Park et al study [20], because it involved a large and homogenous sample, and consequently higher estimates of sensitivity and specificity. After removing two other studies, the estimate of the pooled dOR increases owing to: the Cheng et al study [25] included mostly population at high risk for developing diabetes and considered a cut-off for diagnosing of retinopathy of 5.5% for HbA1c, and therefore provides high sensitivity and low specificity estimates; the Wong et al. study [26] reported low sensitivity estimates including three population-based samples, and excluded participants who had ungradable retinal photographs. A review that analyzed the potential sources of bias and variation in diagnostic accuracy studies, suggested that high variability in the characteristics of participants in the studies testing the accuracy of tests for diabetes retinopathy is significantly associated to lower accuracy estimates [33].

This review has several potential limitations, including publication bias and insufficient information from study reports. Although we found no clear evidence of significant publication bias, studies showing poor test performance might be less (or more) likely to be published. Furthermore, given the high variability in the study results and the fact that most studies used diagnostic cut-offs that differed from the international recommendations, our results must be interpreted with caution. Finally, to ensure that the results can be generalized, we included studies with both diabetic and non-diabetic participants. We expect that antidiabetic medications have the same effect on the HbA1c, FPG and 2h-PG levels; however, we cannot rule out the possibility of some differences associated with specific drugs or clinical settings.

## Conclusion

The three recommended tests for the diagnosis of type 2 diabetes show sufficient accuracy for their use in clinical settings, although the overall accuracy for the diagnosis of retinopathy was slightly higher for HbA1c and 2h-PG than for FPG. Due to the variability and inconveniences of the glucose level-based methods, the HbA1c test might be the most appropriate method for the diagnosis of type 2 diabetes in nonpregnant adults. However, the appropriate use of this information requires an evaluation of the clinical context, specifically, whether the test will be used for screening or diagnosis, the availability of the test in underdeveloped countries and the costs.

## Supporting Information

S1 File **Table A in S1 File. PRISMA Guidelines Checklist. Table B in S1 File QUADAS-2 risk of bias assessment**. U: unclear; Y: yes; N: no; L: low; H: high; HbA1c: glycated haemoglobin; FPG: fasting plasma glucose; 2h-PG: 2-hour plasma glucose. **Table C in S1 File. Subgroup analysis of the four studies that included measurements of HbA1c, FPG and 2h-PG.** Values in parentheses are 95% confidence intervals. FPG: fasting plasma glucose, PLR: positive likelihood ratio, NLR: negative likelihood ratio, dOR: diagnostic odds ratio, AUC: area under receiver operating characteristic curve. **Figure A in S1 File. Quality Assessment of Diagnostic Accuracy Studies (QUADAS-2) criteria, for the reviewed studies. Figure B in S1 File. Forest plot of the sensitivity of each index test for diagnosing diabetes in the reviewed studies.** CI: confidence interval; (a), (b) and (c) indicate different subgroups of participants in that study, as defined by setting (Table 1). **Figure C in S1 File. Forest plot of the specificity of each index test for diagnosing diabetes in the reviewed studies.** CI: confidence interval; (a), (b) and (c) indicate different subgroups of participants in that study, as defined by setting (Table 1). **Figure D in S1 File. Forest plot of the positive likelihood ratio (PLR) of each index test for the diagnosis of diabetes in the reviewed studies.** CI: confidence interval; (a), (b) and (c) indicate different subgroups of participants in that study, as defined by setting (Table 1). **Figure E in S1 File. Forest plot of the negative likelihood ratio (NLR) of each index test for the diagnosis of diabetes in the reviewed studies.** CI: confidence interval; (a), (b) and (c) indicate different subgroups of participants in that study, as defined by setting (Table 1). **Figure F in S1 File. Assessment of potential bias due to including each study in the review, by index test.** dOR: Diagnostic odds ratio; CI: confidence interval; (a), (b) and (c) indicate different subgroups of participants in that study, as defined by setting (Table 1). **Figure G in S1 File. Funnel plot for the assessment of potential publication bias.** ESS: Effective sample size. **References A in S1 File. Studies excluded from the systematic review and meta-analyses and main reasons for their exclusion.**
(DOCX)

## References

[1] The Expert Committee on the Diagnosis and Classification of Diabetes Mellitus. Report of the expert committee on the diagnosis and classification of diabetes mellitus. Diabetes Care 1997;20(7):1183–97. 9203460 10.2337/diacare.20.7.1183

[2] The International Expert Committee. International expert committee report on the role of the A1C assay in the diagnosis of diabetes. Diabetes Care 2009;32(7):1327–34. 10.2337/dc09-9033 19502545 PMC2699715

[3] American Diabetes Association. (2) Classification and diagnosis of diabetes. Diabetes Care 2015;38(Supplement 1):S8–16. 10.2337/dc15-S005 25537714

[4] World Health Organization. Use of glycated haemoglobin (HbA1c) in the diagnosis of diabetes mellitus: abbreviated report of a WHO consultation. Geneva: WHO; 2011. 26158184

[5] Bonora E, Tuomilehto J. The pros and cons of diagnosing diabetes with A1 C. Diabetes Care 2011;34(Supplement 2):S184–90. 10.2337/dc11-s216 PMC3632159 21525453

[6] Nathan DM. Diabetes advances in diagnosis and treatment. JAMA 2015; 314(10):1052–1062. 10.1001/jama.2015.9536 26348754

[7] Fizelova M, Stančáková A, Lorenzo C, Haffner SM, Cederberg H, Kuusisto J, et al Glycated hemoglobin levels are mostly dependent on nonglycemic parameters in 9398 Finnish men without diabetes. Journal of Clinical Endocrinology and Metabolism 2015;100(5):1989–96. 10.1210/jc.2014-4121 25734252

[8] Malkani S, Mordes JP. Implications of using hemoglobin A1C for diagnosing diabetes mellitus. The American Journal of Medicine 2011;124(5):395–401. 10.1016/j.amjmed.2010.11.025 21531226 PMC3086708

[9] Colagiuri S, Lee CM, Wong TY, Balkau B, Shaw JE, Borch-Johnsen K. Glycemic thresholds for diabetes-specific retinopathy: implications for diagnostic criteria for diabetes. Diabetes Care 2011;34(1):145–50. 10.2337/dc10-1206 20978099 PMC3005450

[10] QUADAS-2 Group. QUADAS-2: a revised tool for the quality assessment of diagnostic accuracy studies. Annals of Internal Medicine 2011;155(8):529–36. 10.7326/0003-4819-155-8-201110180-00009 22007046

[11] Moher D, Liberati A, Tetzlaff J, Altman DG. Preferred reporting items for systematic reviews and meta-analyses: the PRISMA statement. International Journal of Surgery 2010;8(5):336–41. 10.1016/j.ijsu.2010.02.007 20171303

[12] Macaskill P, Gatsonis C, Deeks J, Harbord R, Takwoingi Y. Cochrane handbook for systematic reviews of diagnostic test accuracy. Version 09 0 London: The Cochrane Collaboration; 2010.

[13] Lijmer JG, Bossuyt PM, Heisterkamp SH. Exploring sources of heterogeneity in systematic reviews of diagnostic tests. Statistics in medicine 2002;21(11), 1525–1537. 12111918 10.1002/sim.1185

[14] Jones CM, Athanasiou T. Summary receiver operating characteristic curve analysis techniques in the evaluation of diagnostic tests. The Annals of Thoracic Surgery 2005;79(1):16–20. 15620907 10.1016/j.athoracsur.2004.09.040

[15] Reitsma JB, Glas AS, Rutjes AW, Scholten RJ, Bossuyt PM, Zwinderman AH. Bivariate analysis of sensitivity and specificity produces informative summary measures in diagnostic reviews. Journal of Clinical Epidemiology 2005;58(10):982–90. 16168343 10.1016/j.jclinepi.2005.02.022

[16] Higgins JP, Thompson SG. Quantifying heterogeneity in a meta-analysis. Statistics in Medicine 2002;21(11):1539–58. 12111919 10.1002/sim.1186

[17] Deeks JJ, Macaskill P, Irwig L. The performance of tests of publication bias and other sample size effects in systematic reviews of diagnostic test accuracy was assessed. Journal of Clinical Epidemiology 2005;58(9):882–93. 16085191 10.1016/j.jclinepi.2005.01.016

[18] Sabanayagam C, Khoo EY, Lye WK, Ikram MK, Lamoureux EL, Cheng CY, et al Diagnosis of diabetes mellitus using HbA1c in Asians: relationship between HbA1c and retinopathy in a multiethnic Asian population. The Journal of Clinical Endocrinology and Metabolism 2015;100(2):689–96. 10.1210/jc.2014-2498 25375980

[19] Mukai N, Yasuda M, Ninomiya T, Hata J, Hirakawa Y, Ikeda F, et al Thresholds of various glycemic measures for diagnosing diabetes based on prevalence of retinopathy in community-dwelling Japanese subjects: the Hisayama Study. Cardiovascular Diabetology 2014;13(1):45. 24533962 10.1186/1475-2840-13-45 PMC3936993

[20] Park YM, Ko SH, Lee JM, Kim DJ, Kim DJ, Han K, et al Glycaemic and haemoglobin A1c thresholds for detecting diabetic retinopathy: the fifth Korea National Health and Nutrition Examination Survey (2011). Diabetes Research and Clinical Practice 2014;104(3):435–42. 10.1016/j.diabres.2014.04.003 24785739

[21] Cho NH, Kim TH, Woo SJ, Park KH, Lim S, Cho YM, et al Optimal HbA1c cutoff for detecting diabetic retinopathy. Acta Diabetologica 2013;50(6):837–42. 10.1007/s00592-013-0452-3 23354926

[22] Xin Z, Yuan MX, Li HX, Hua L, Feng JP, Shi J, et al Evaluation for fasting and 2-hour glucose and HbA1c for diagnosing diabetes based on prevalence of retinopathy in a Chinese population. PLoS One 2012;7(7):e40610 10.1371/journal.pone.0040610 22808204 PMC3395674

[23] Massin P, Lange C, Tichet J, Erginay A, Cailleau M, Eschwege E, et al; DESIR Study Group. Hemoglobin A1c and fasting plasma glucose levels as predictors of retinopathy at 10 years: the French DESIR study. Archives of Ophthalmology 2011;129(2):188–95. 10.1001/archophthalmol.2010.353 21320965 PMC3317887

[24] Jonas JB, Xu L, Xie XW, Wang YX. Relationship between fasting glucose and retinopathy for diagnosis of diabetes: results from a population-based study in urban and rural China. Retina 2010;30(8):1223–7. 20827140 10.1097/IAE.0b013e3181ce74ae

[25] Cheng YJ, Gregg EW, Geiss LS, Imperatore G, Williams DE, Zhang X, et al Association of A1C and fasting plasma glucose levels with diabetic retinopathy prevalence in the U.S. population: Implications for diabetes diagnostic thresholds. Diabetes Care 2009;32(11):2027–32. 10.2337/dc09-0440 19875604 PMC2768189

[26] Wong TY, Liew G, Tapp RJ, Schmidt MI, Wang JJ, Mitchell P, et al Relation between fasting glucose and retinopathy for diagnosis of diabetes: three population-based cross-sectional studies. Lancet 2008;371(9614):736–43. 10.1016/S0140-6736(08)60343-8 18313502 PMC2350208

[27] Miyazaki M, Kubo M, Kiyohara Y, Okubo K, Nakamura H, Fujisawa K, et al Comparison of diagnostic methods for diabetes mellitus based on prevalence of retinopathy in a Japanese population: the Hisayama Study. Diabetologia 2004;47(8):1411–5. 15309291 10.1007/s00125-004-1466-8

[28] McCance DR, Hanson RL, Charles MA, Jacobsson LT, Pettitt DD, Bennett PH, et al Comparison of tests for glycated haemoglobin and fasting and two hour plasma glucose concentrations as diagnostic methods for diabetes. BMJ 1994;308(6940):1323–8. 8019217 10.1136/bmj.308.6940.1323 PMC2540244

[29] Bennett CM, Jenkins AJ, Guo M, Dharmage SC. Could global standardization of A1C help it become the preferred diabetes screening method? Review of Endocrinology 2007 6:59–61.

[30] Diabetes Prevention Program Research Group. The prevalence of retinopathy in impaired glucose tolerance and recent-onset diabetes in the Diabetes Prevention Program. Diabetic Medicine 2007;24(2):137–44. 17257275 10.1111/j.1464-5491.2007.02043.x PMC2267935

[31] Charoensawat S, Böhning W, Böhning D, Holling H. Meta-analysis and meta-modelling for diagnostic problems. BMC medical research methodology 2014; 14(1), 1. 24758534 10.1186/1471-2288-14-56 PMC4007022

[32] Plana MN, Abraira V, Zamora J. An Introduction to Diagnostic Meta-analysis Methods of Clinical Epidemiology: Springer; 2013: 103–20. Berlin Heidelberg.

[33] Rutjes AW, Reitsma JB, Di Nisio M, Smidt N, Van Rijn JC, Bossuyt PM. Evidence of bias and variation in diagnostic accuracy studies. Canadian Medical Association Journal 2006; 174(4), 469–476. 16477057 10.1503/cmaj.050090 PMC1373751

