# Supplementary material for: The Accuracy of Diagnostic Methods for Diabetic Retinopathy: A Systematic Review and Meta-Analysis
Source: PLoS One. 2016 Apr 28;11(4):e0154411. doi: 10.1371/journal.pone.0154411 (PMC4849768; doi:10.1371/journal.pone.0154411)
Supplement: S1 File — Table A in S1 File. PRISMA Guidelines Checklist. Table B in S1 File QUADAS-2 risk of bias assessment. U: unclear; Y: yes; N: no; L: low; H: high; HbA1c: glycated haemoglobin; FPG: fasting plasma glucose; 2h-PG: 2-hour plasma glucose. Table C in S1 File. Subgroup analysis of the four studies that included measurements of HbA1c, FPG and 2h-PG. Values in parentheses are 95% confidence intervals. FPG: fasting plasma glucose, PLR: positive likelihood ratio, NLR: negative likelihood ratio, dOR: diagnostic odds ratio, AUC: area under receiver operating characteristic curve. Figure A in S1 File. Quality Assessment of Diagnostic Accuracy Studies (QUADAS-2) criteria, for the reviewed studies. Figure B in S1 File. Forest plot of the sensitivity of each index test for diagnosing diabetes in the reviewed studies. CI: confidence interval; (a), (b) and (c) indicate different subgroups of participants in that study, as defined by setting (Table 1). Figure C in S1 File. Forest plot of the specificity of each index test for diagnosing diabetes in the reviewed studies. CI: confidence interval; (a), (b) and (c) indicate different subgroups of participants in that study, as defined by setting (Table 1). Figure D in S1 File. Forest plot of the positive likelihood ratio (PLR) of each index test for the diagnosis of diabetes in the reviewed studies. CI: confidence interval; (a), (b) and (c) indicate different subgroups of participants in that study, as defined by setting (Table 1). Figure E in S1 File. Forest plot of the negative likelihood ratio (NLR) of each index test for the diagnosis of diabetes in the reviewed studies. CI: confidence interval; (a), (b) and (c) indicate different subgroups of participants in that study, as defined by setting (Table 1). Figure F in S1 File. Assessment of potential bias due to including each study in the review, by index test. dOR: Diagnostic odds ratio; CI: confidence interval; (a), (b) and (c) indicate different subgroups of participants in that study [file pone.0154411.s001.docx]

**S1 File**

**Table A** PRISMA Guidelines Checklist

| **Section/topic** | **#** | **Checklist item** | **Reported on page #** |
| --- | --- | --- | --- |
| **TITLE** | | |  |
| Title | 1 | Identify the report as a systematic review, meta-analysis, or both. | 1 |
| **ABSTRACT** | | |  |
| Structured summary | 2 | Provide a structured summary including, as applicable: background; objectives; data sources; study eligibility criteria, participants, and interventions; study appraisal and synthesis methods; results; limitations; conclusions and implications of key findings; systematic review registration number. | 2-3 |
| **INTRODUCTION** | | |  |
| Rationale | 3 | Describe the rationale for the review in the context of what is already known. | 3-5 |
| Objectives | 4 | Provide an explicit statement of questions being addressed with reference to participants, interventions, comparisons, outcomes, and study design (PICOS). | 5 |
| **METHODS** | | |  |
| Protocol and registration | 5 | Indicate if a review protocol exists, if and where it can be accessed (e.g., Web address), and, if available, provide registration information including registration number. | N/A |
| Eligibility criteria | 6 | Specify study characteristics (e.g., PICOS, length of follow-up) and report characteristics (e.g., years considered, language, publication status) used as criteria for eligibility, giving rationale. | 5-6 |
| Information sources | 7 | Describe all information sources (e.g., databases with dates of coverage, contact with study authors to identify additional studies) in the search and date last searched. | 5 |
| Search | 8 | Present full electronic search strategy for at least one database, including any limits used, such that it could be repeated. | 5 |
| Study selection | 9 | State the process for selecting studies (i.e., screening, eligibility, included in systematic review, and, if applicable, included in the meta-analysis). | 6 |
| Data collection process | 10 | Describe method of data extraction from reports (e.g., piloted forms, independently, in duplicate) and any processes for obtaining and confirming data from investigators. | 6 |
| Data items | 11 | List and define all variables for which data were sought (e.g., PICOS, funding sources) and any assumptions and simplifications made. | 6 |
| Risk of bias in individual studies | 12 | Describe methods used for assessing risk of bias of individual studies (including specification of whether this was done at the study or outcome level), and how this information is to be used in any data synthesis. | 6 |
| Summary measures | 13 | State the principal summary measures (e.g., risk ratio, difference in means). | 7 |
| Synthesis of results | 14 | Describe the methods of handling data and combining results of studies, if done, including measures of consistency (e.g., I^2^) for each meta-analysis. | 7-8 |
| Risk of bias across studies | 15 | Specify any assessment of risk of bias that may affect the cumulative evidence (e.g., publication bias, selective reporting within studies). | 8 |
| Additional analyses | 16 | Describe methods of additional analyses (e.g., sensitivity or subgroup analyses, meta-regression), if done, indicating which were pre-specified. | 8 |
| **RESULTS** | | |  |
| Study selection | 17 | Give numbers of studies screened, assessed for eligibility, and included in the review, with reasons for exclusions at each stage, ideally with a flow diagram. | 8 |
| Study characteristics | 18 | For each study, present characteristics for which data were extracted (e.g., study size, PICOS, follow-up period) and provide the citations. | 8-9 |
| Risk of bias within studies | 19 | Present data on risk of bias of each study and, if available, any outcome level assessment (see item 12). | 9 |
| Results of individual studies | 20 | For all outcomes considered (benefits or harms), present, for each study: (a) simple summary data for each intervention group (b) effect estimates and confidence intervals, ideally with a forest plot. | 9-10 |
| Synthesis of results | 21 | Present results of each meta-analysis done, including confidence intervals and measures of consistency. | 9-10 |
| Risk of bias across studies | 22 | Present results of any assessment of risk of bias across studies (see Item 15). | 10 |
| Additional analysis | 23 | Give results of additional analyses, if done (e.g., sensitivity or subgroup analyses, meta-regression [see Item 16]). | 10 |
| **DISCUSSION** | | |  |
| Summary of evidence | 24 | Summarize the main findings including the strength of evidence for each main outcome; consider their relevance to key groups (e.g., healthcare providers, users, and policy makers). | 11-13 |
| Limitations | 25 | Discuss limitations at study and outcome level (e.g., risk of bias), and at review-level (e.g., incomplete retrieval of identified research, reporting bias). | 13 |
| Conclusions | 26 | Provide a general interpretation of the results in the context of other evidence, and implications for future research. | 13-14 |
| **FUNDING** | | |  |
| Funding | 27 | Describe sources of funding for the systematic review and other support (e.g., supply of data); role of funders for the systematic review. | N/A |

**Table B** QUADAS-2 risk of bias assessment

|  | **Sabanayagam 2015** | **Mukai 2014** | **Park 2014** | **Cho 2013** | **Xin 2012** | **Massin 2011** | **Jonas 2010** | **Cheng 2009** | **Wong 2008** | **Miyazaki 2004** | **McCance 1994** |
| --- | --- | --- | --- | --- | --- | --- | --- | --- | --- | --- | --- |
| **DOMAIN 1: PATIENT SELECTION** |  |  |  |  |  |  |  |  |  |  |  |
| Was a consecutive or random sample of patients enrolled? | **U** | **Y** | **U** | **Y** | **Y** | **Y** | **U** | **Y** | **U** | **Y** | **U** |
| Was a case-­‐control design avoided? | **Y** | **Y** | **U** | **Y** | **Y** | **Y** | **U** | **Y** | **Y** | **Y** | **Y** |
| Did the study avoid inappropriate exclusions? | **U** | **Y** | **Y** | **Y** | **Y** | **Y** | **N** | **Y** | **Y** | **Y** | **N** |
| **Risk of Bias** | **U** | **L** | **U** | **L** | **L** | **L** | **U** | **L** | **U** | **L** | **U** |
| **Concerns regarding applicability** | **U** | **L** | **L** | **L** | **L** | **L** | **H** | **L** | **L** | **L** | **U** |
|  |  |  |  |  |  |  |  |  |  |  |  |
| **DOMAIN 2: INDEX TEST(S)** | **HbA1c** | **HbA1c/ FPG/ 2h-PG** | **HbA1c/ FPG** | **HbA1c/ FPG** | **HbA1c/ FPG/ 2h-PG** | **HbA1c/ FPG** | **FPG** | **HbA1c/ FPG** | **FPG** | **HbA1c/ FPG/ 2h-PG** | **HbA1c/ FPG/ 2h-PG** |
| Were the index test results interpreted without knowledge of the results of the reference standard? | **Y** | **Y/ Y/ Y** | **Y/ Y** | **Y/ N** | **Y/ Y /Y** | **N/ N** | **Y** | **Y/ Y** | **Y** | **Y/ Y/ Y** | **N/ N / N** |
| If a threshold was used, was it pre-­‐specified? | **Y** | **N/ N/ N** | **N/ N** | **N/ N** | **N/ N/ N** | **N/ N** | **N** | **N/ N** | **Y** | **N/ N/ N** | **N/ N/ N** |
| **Risk of Bias** | **L** | **L/ L /L** | **L/ L** | **L/ H** | **L/ L/ L** | **H/ H** | **L** | **L/ L** | **L** | **L/ L/ L** | **H/ H/ H** |
| **Concerns regarding applicability** | **L** | **U/ U/ U** | **U/ U** | **U/ H** | **U/ U/ U** | **H/ H** | **U** | **U/ U** | **L** | **U/ U/ U** | **H/ H/ H** |
|  |  |  |  |  |  |  |  |  |  |  |  |
| **DOMAIN 3: REFERENCE STANDARD** |  |  |  |  |  |  |  |  |  |  |  |
| Is the reference standard likely to correctly classify the target condition? | **Y** | **Y** | **Y** | **Y** | **Y** | **Y** | **Y** | **Y** | **Y** | **Y** | **Y** |
| Were the reference standard results interpreted without knowledge of the results of the index test? | **Y** | **Y** | **Y** | **Y** | **Y** | **Y** | **Y** | **Y** | **Y** | **Y** | **Y** |
| **Risk of Bias** | **L** | **L** | **L** | **L** | **L** | **L** | **L** | **L** | **L** | **L** | **L** |
| **Concerns regarding applicability** | **L** | **L** | **L** | **L** | **L** | **L** | **L** | **L** | **L** | **L** | **L** |
|  |  |  |  |  |  |  |  |  |  |  |  |
| **DOMAIN 4: FLOW AND TIMING** |  |  |  |  |  |  |  |  |  |  |  |
| Was there an appropriate interval between index test(s) and reference standard? | **Y** | **Y** | **Y** | **Y** | **Y** | **Y** | **Y** | **Y** | **Y** | **Y** | **Y** |
| Did all patients receive a reference standard? | **N** | **N** | **N** | **N** | **N** | **N** | **Y** | **N** | **N** | **N** | **N** |
| Did patients receive the same reference standard? | **Y** | **Y** | **Y** | **Y** | **Y** | **Y** | **Y** | **Y** | **Y** | **Y** | **Y** |
| Were all patients included in the analysis? | **N** | **N** | **N** | **N** | **N** | **N** | **N** | **N** | **N** | **N** | **N** |
| **Risk of Bias** | **L** | **L** | **L** | **L** | **L** | **L** | **L** | **L** | **L** | **L** | **L** |

U: unclear; Y: yes; N: no; L: low; H: high; HbA1c: glycated haemoglobin; FPG: fasting plasma glucose; 2h-PG: 2-hour plasma glucose.

**Table C** Subgroup analysis of the four studies that included measurements of HbA1c, FPG and 2h-PG.

|  | **Sensitivity (%)** | **Specificity (%)** | **PLR** | **NLR** | **dOR** | **AUC** |
| --- | --- | --- | --- | --- | --- | --- |
| HbA1c | 83.0 (71.0–97.0) | 89.0 (87.0–91.0) | 7.23 (2.31–22.56) | 0.19 (0.06–0.59) | 34.68 (23.56–51.03) | 0.882 (0.835–0.930) |
| FPG | 79.0 (67.0–92.0) | 87.0 (85.0–89.0) | 6.22 (1.95–19.87) | 0.23 (0.07–0.72) | 24.79 (17.40–35.32) | 0.868 (0.824–0.912) |
| 2h-PG | 82.0 (75.0–91.0) | 86.0 (85.0–87.0) | 7.44 (2.42–22.87) | 0.17 (0.06–0.53) | 32.39 (25.27–41.51) | 0.916 (0.870–0.963) |

Values in parentheses are 95% confidence intervals. FPG: fasting plasma glucose, PLR: positive likelihood ratio, NLR: negative likelihood ratio, dOR: diagnostic odds ratio, AUC: area under receiver operating characteristic curve.

**Figure A** Quality Assessment of Diagnostic Accuracy Studies (QUADAS-2) criteria, for the reviewed studies.

**Figure B** Forest plot of the sensitivity of each index test for diagnosing diabetes in the reviewed studies.

**
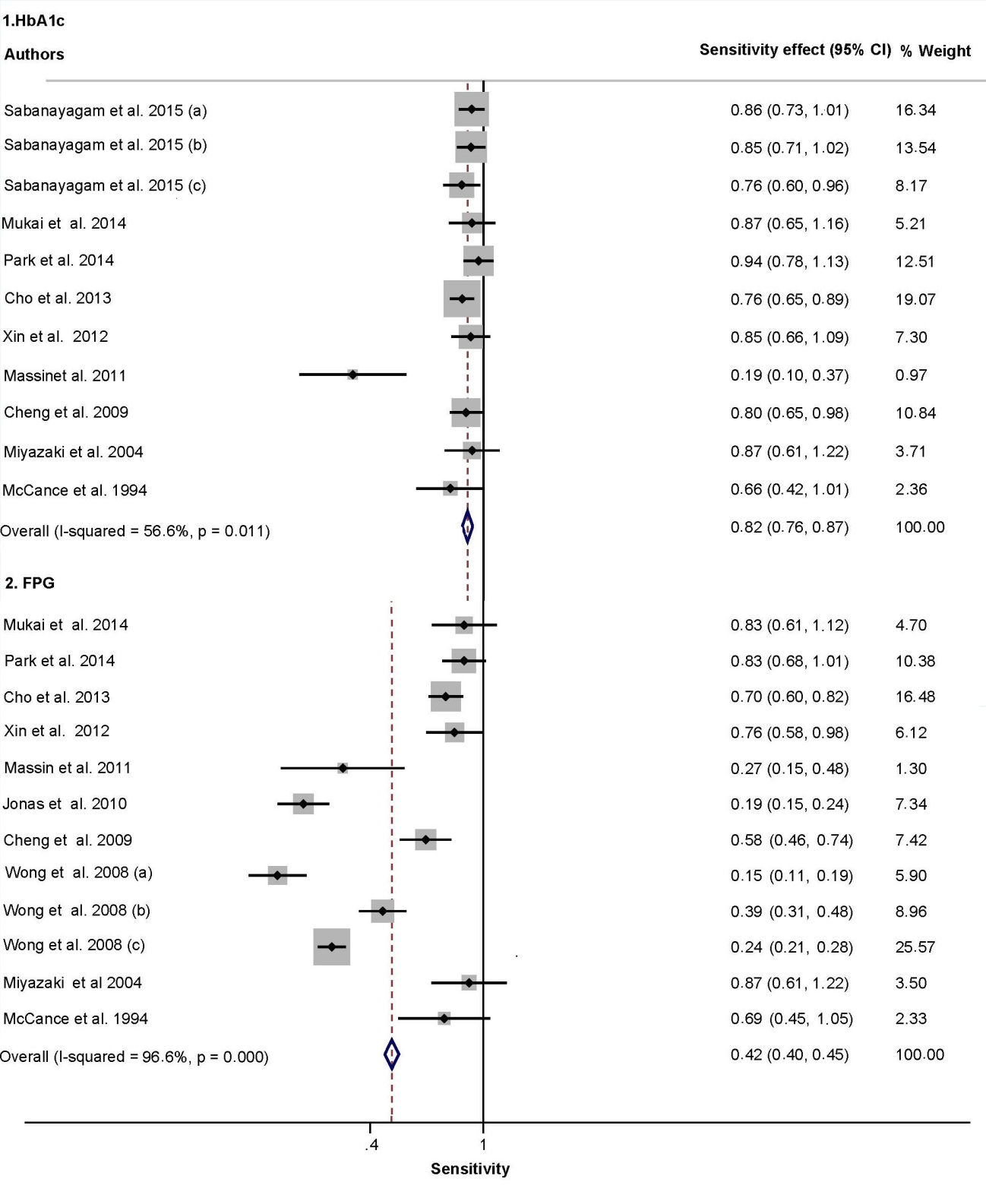
**

CI: confidence interval; (a), (b) and (c) indicate different subgroups of participants in that study, as defined by setting (Table 1).

**Figure C** Forest plot of the specificity of each index test for diagnosing diabetes in the reviewed studies.

**
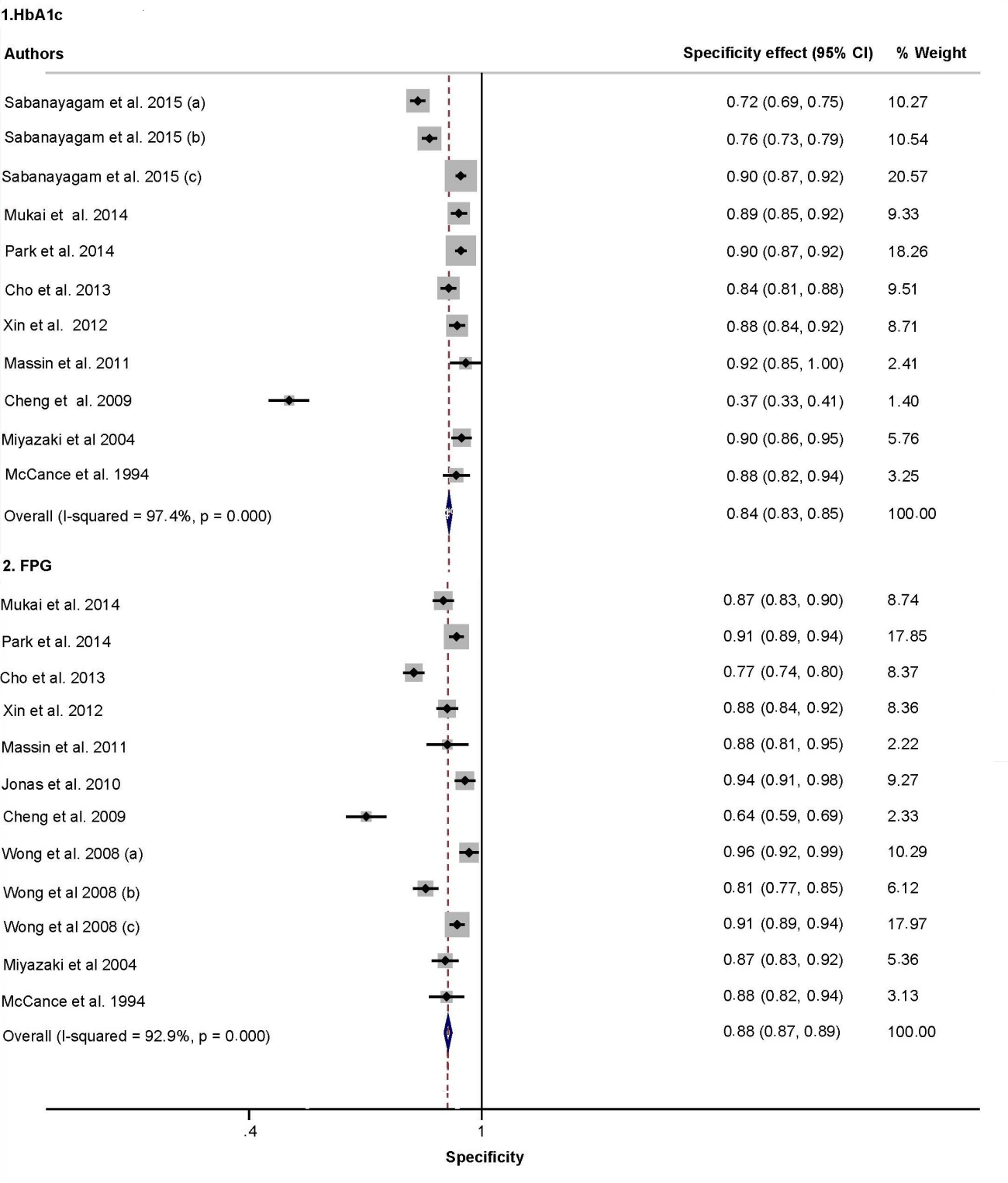
**

CI: confidence interval; (a), (b) and (c) indicate different subgroups of participants in that study, as defined by setting (Table 1).

**Figure D** Forest plot of the positive likelihood ratio (PLR) of each index test for the diagnosis of diabetes in the reviewed studies.

**
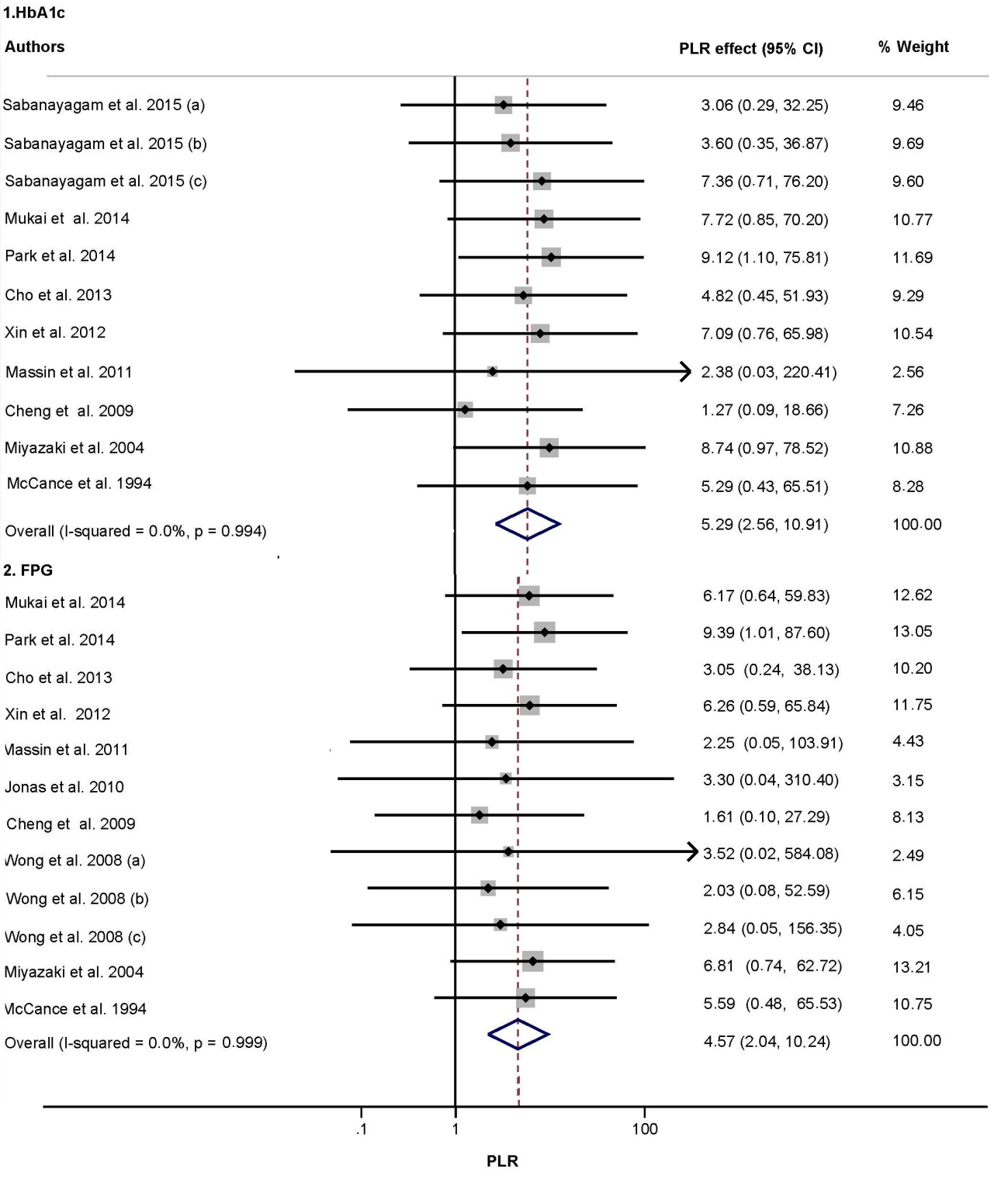
**

CI: confidence interval; PLR: positive likelihood ratio; (a), (b) and (c) indicate different subgroups of participants in that study, as defined by setting (Table 1).

**Figure E** Forest plot of the negative likelihood ratio (NLR) of each index test for the diagnosis of diabetes in the reviewed studies.


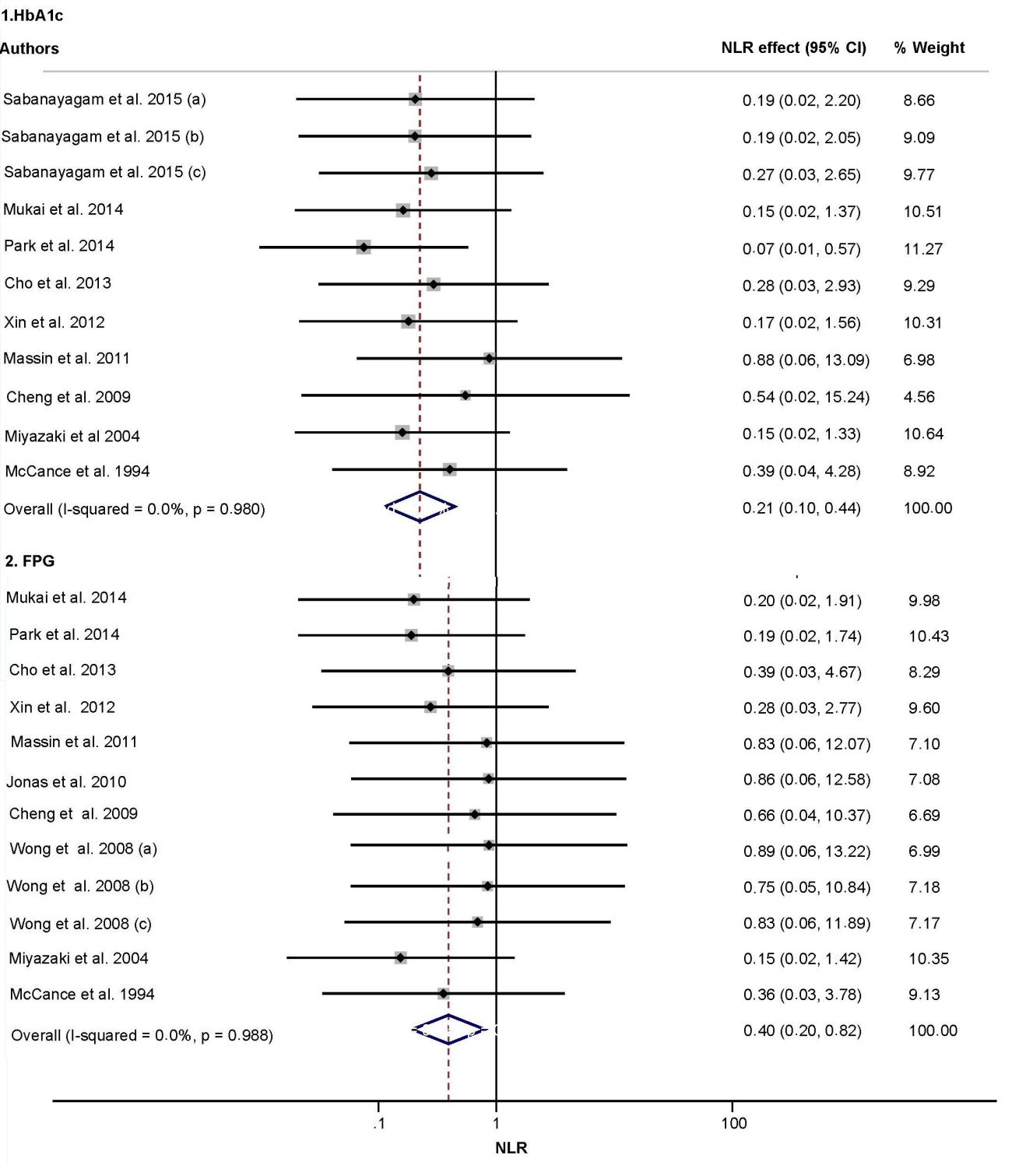


CI: confidence interval; NLR: negative likelihood ratio; (a), (b) and (c) indicate different subgroups of participants in that study, as defined by setting (Table 1).

**Figure F** Assessment of potential bias due to including each study in the review, by index test.


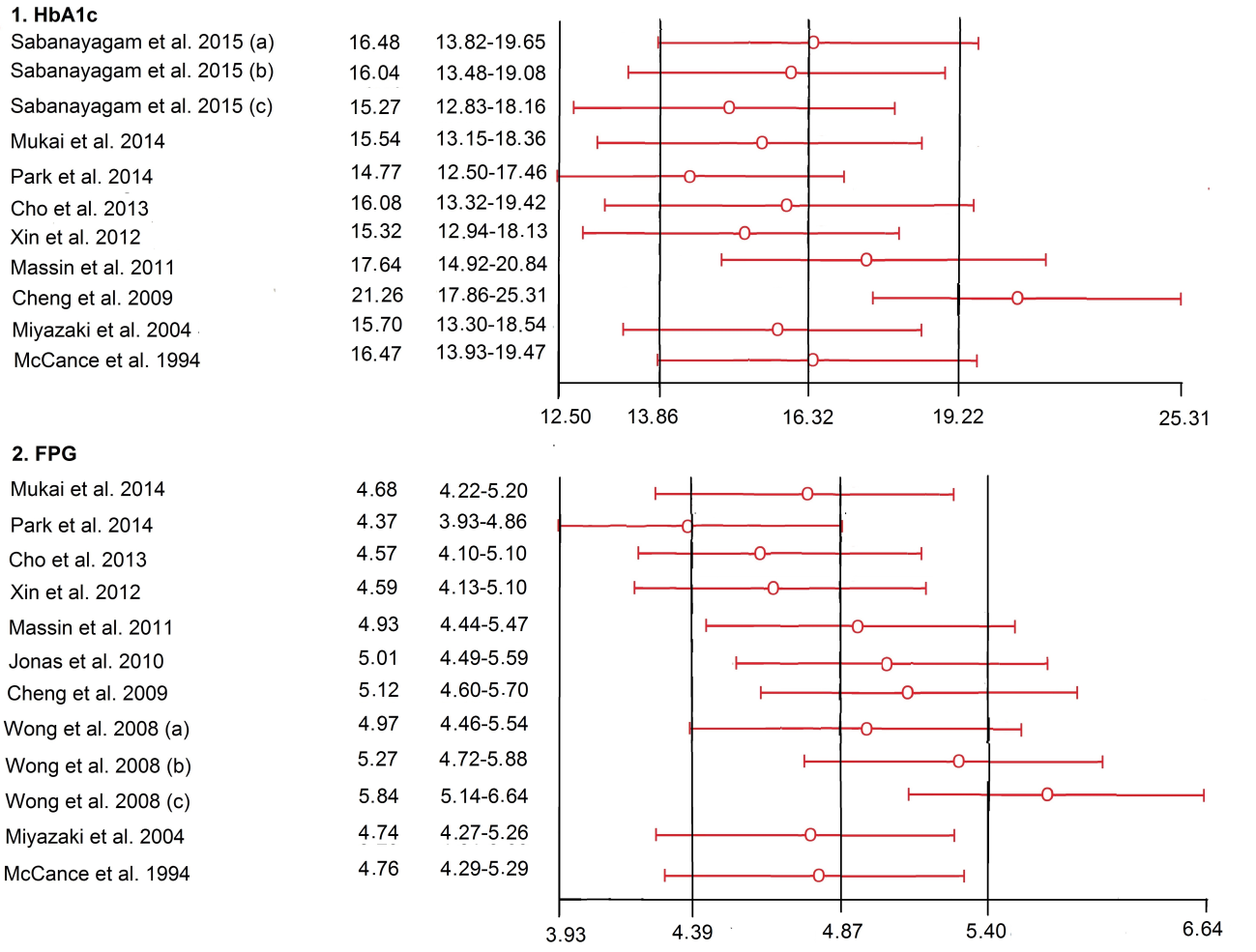


dOR: Diagnostic odds ratio; CI: confidence interval; (a),(b) and (c) indicate different subgroups of participants in that study, as defined by setting (Table 1).

**Figure G** Funnel plot for the assessment of potential publication bias. ESS: Effective sample size.

**
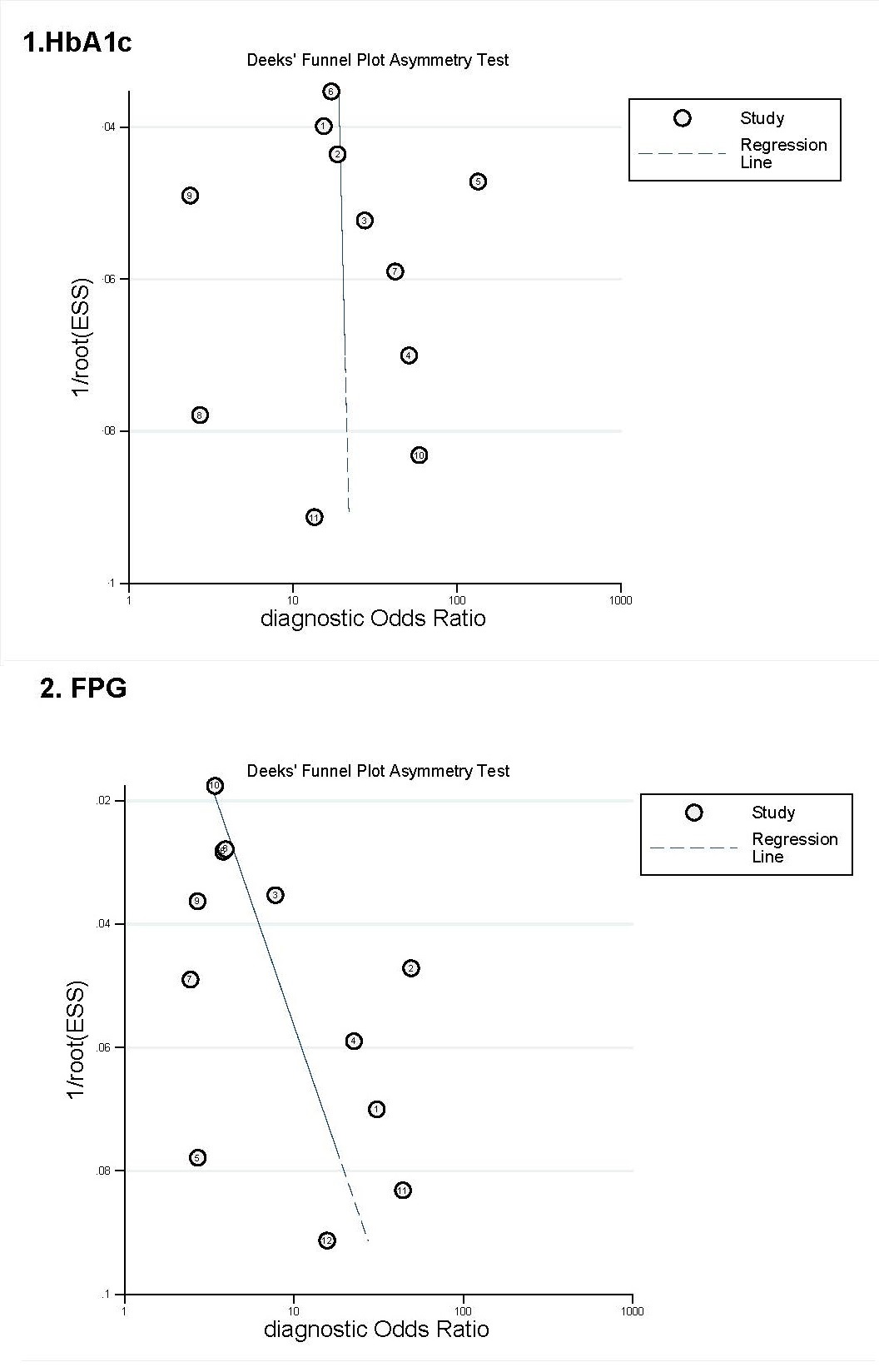
**

**References A** Studies excluded from the systematic review and meta-analyses and main reasons for their exclusion.

Almdal TP, Handlos LN, Valerius M, et al. Glycaemic threshold for diabetes-specific retinopathy among individuals from Saudi Arabia, Algeria and Portugal. Diabetes Research and Clinical Practice 2014;103(3):e44–6. [Does not have sufficient data to reconstruct 2 × 2 table]

Chen P, Ong RTH, Tay WT, et al. A study assessing the association of glycated hemoglobin A_1C_ (HbA_1C_) associated variants with HbA_1C_, chronic kidney disease and diabetic retinopathy in populations of Asian ancestry. PLoS ONE 2013;8(11):e79767. doi:10.1371/journal.pone.0079767 [Does not fulfil eligibility or methodological criteria]

Cheng YJ, Gregg EW, Narayan KMV, et al. Fasting and 2-hour glucose and glycated hemoglobin levels and retinopathy in U.S. adults: searching for a threshold. In Diabetes 2006;55:A207–8. [Is not a scientific article]

Choi SH, Kim TH, Lim S, et al. Hemoglobin A_1c_ as a diagnostic tool for diabetes screening and new-onset diabetes prediction: a 6-year community-based prospective study. Diabetes Care 2011;34(4):944–9. [Does not fulfil eligibility or methodological criteria]

Colagiuri S, Lee CMY, Wong TY, et al. Glycemic thresholds for diabetes-specific retinopathy implications for diagnostic criteria for diabetes. Diabetes Care 2011;34(1):145–50. [Does not have sufficient data to reconstruct 2 × 2 table]

Doi Y, Kubo M, Yonemoto K, et al. Fasting plasma glucose cutoff for diagnosis of diabetes in a Japanese population. The Journal of Clinical Endocrinology and Metabolism 2008;93(9):3425–9. [Does not fulfil eligibility or methodological criteria]

Eid WE, Pottala JV. Value of hemoglobin A1c in diagnosing diabetes mellitus within a chronic disease management system illustrated by the receiver operating characteristic curve. Endocrine Practice 2010;16(1):14–20. [Does not fulfil eligibility or methodological criteria]

Engelgau MM, Thompson TJ, Herman WH, et al. Comparison of fasting and 2-hour glucose and HbA1c levels for diagnosing diabetes: diagnostic criteria and performance revisited. Diabetes Care 1997;20(5):785–91. [Does not have sufficient data to reconstruct 2 × 2 table]

Ferrannini E, Massari M, Nannipieri M, et al. Plasma glucose levels as predictors of diabetes: the Mexico City diabetes study. Diabetologia 2009;52(5):818–24. [Does not fulfil eligibility or methodological criteria]

Franco LJ, Dal Fabbro AL, Martinez EZ, et al. Performance of glycated haemoglobin (HbA1c) as a screening test for diabetes and impaired glucose tolerance (IGT) in a high risk population—The Brazilian Xavante Indians. Diabetes Research and Clinical Practice 2014;106(2):337–42. [Does not fulfil eligibility or methodological criteria]

Ginde AA, Cagliero E, Nathan DM, et al. Value of risk stratification to increase the predictive validity of HbA1c in screening for undiagnosed diabetes in the US population. Journal of General Internal Medicine 2008;23(9):1346–53. [Does not fulfil eligibility or methodological criteria]

Gnaneswaran S, Kuberan D, Vinodhini VM, et al. Fasting plasma glucose and glycated hemoglobin in the prediction of diabetic retinopathy in a rural population. International Journal of Pharmaceutical and Clinical Research 2014;6(1):40–5. [Does not have sufficient data to reconstruct 2 × 2 table]

Herdzik E, Safranow K, Ciechanowski K. Diagnostic value of fasting capillary glucose, fructosamine and glycosylated haemoglobin in detecting diabetes and other glucose tolerance abnormalities compared to oral glucose tolerance test. Acta Diabetologica 2002;39(1):15–22. [Does not fulfil eligibility or methodological criteria]

Isnard F, Eschwège E. Vascular complications of diabetes: is there a glycemic threshold? La Revue du Praticien 2001;51(16):1759–64. [Not available in English]

Ito C, Maeda R, Ishida S, et al. Importance of OGTT for diagnosing diabetes mellitus based on prevalence and incidence of retinopathy. Diabetes Research and Clinical Practice 2000;49(2–3):181–6. [Does not have sufficient data to reconstruct 2 × 2 table]

Johnson JL, Duick DS, Chui MA, et al. Identifying prediabetes using fasting insulin levels. Endocrine Practice 2010;16(1):47–52. [Does not fulfil eligibility or methodological criteria]

Jung JH, Kim ST, Cho YZ, et al. Acceptability of HbA1c values as a diagnostic tool for diabetes mellitus in Korea. The Korean Journal of Medicine 2010;79(6):673–80. [Not available in English]

Kim JM, Kim DJ. The optimal cutoff value of glycated hemoglobin for detection of diabetic retinopathy. Diabetes & Metabolism Journal 2015;39(1):16–26. [Review]

Kowall B, Rathmann W. HbA_1c_ for diagnosis of type 2 diabetes. Is there an optimal cut point to assess high risk of diabetes complications, and how well does the 6.5% cutoff perform? Diabetes, Metabolic Syndrome and Obesity: Targets and Therapy 2013;6:477–91. [Review]

Krakoff J, Hanson RL, Kobes S, et al. Comparison of the effect of plasma glucose concentrations on microvascular disease between Pima Indian youths and adults. Diabetes Care 2001;24(6):1023–8. [Shared sample]

Lee H, Oh JY, Sung YA, et al. Optimal hemoglobin A1C cutoff value for diagnosing type 2 diabetes mellitus in Korean adults. Diabetes Research and Clinical Practice 2013;99(2):231–6. [Does not fulfil eligibility or methodological criteria]

Liew G, Wong TY. Fasting glucose and retinopathy as a diabetes diagnosis. Retina Today 2008 Sep/Oct:66–70. [Shared sample]

Lipscombe L. HbA1c levels had low sensitivity but high specificity for screening for diabetes. Annals of Internal Medicine 2011;154(8):JC4–9. [Does not fulfil eligibility or methodological criteria]

López-Jaramillo P, Velandia-Carrillo C, Gómez-Arbeláez D, et al. Is the present cut-point to define type 2 diabetes appropriate in Latin-Americans? World Journal of Diabetes 2014;5(6):747–55. [Does not fulfil eligibility or methodological criteria]

Maple‐Brown LJ, Ye C, Retnakaran R. Area‐under‐the‐HbA1c‐curve above the normal range and the prediction of microvascular outcomes: an analysis of data from the Diabetes Control and Complications Trial. Diabetic Medicine 2013;30(1):95–9. [Does not have sufficient data to reconstruct 2 × 2 table]

Marley JV, Oh MS, Hadgraft N, et al. Cross-sectional comparison of point-of-care with laboratory HbA_1c_ in detecting diabetes in real-world remote Aboriginal settings. BMJ Open 2015;5(3):e006277. doi: 10.1136/bmjopen-2014-006277. [Does not fulfil eligibility or methodological criteria]

Mohan V, Vijayachandrika V, Gokulakrishnan K, et al. A1C cut points to define various glucose intolerance groups in Asian Indians. Diabetes Care 2010;33(3):515–9. [Does not fulfil eligibility or methodological criteria]

Mostafa SA, Khunti K, Kilpatrick ES, et al. Diagnostic performance of using one- or two-HbA1c cut-point strategies to detect undiagnosed type 2 diabetes and impaired glucose regulation within a multi-ethnic population. Diabetes and Vascular Disease Research 2013;10(1):84–92. [Does not fulfil eligibility or methodological criteria]

Peter A, Fritsche A, Stefan N, et al. Diagnostic value of hemoglobin A1c for type 2 diabetes mellitus in a population at risk. Experimental and Clinical Endocrinology & Diabetes 2011;119(4):234–7. [Does not fulfil eligibility or methodological criteria]

Rajala U, Laakso M, Qiao Q, et al. Prevalence of retinopathy in people with diabetes, impaired glucose tolerance, and normal glucose tolerance. Diabetes Care 1998;21(10):1664–9. [Does not have sufficient data to reconstruct 2 × 2 table]

Raman R, Verma A, Pal SS, et al. Influence of glycosylated hemoglobin on sight-threatening diabetic retinopathy: a population-based study. Diabetes Research and Clinical Practice 2011;92(2):168–73. [Does not fulfil eligibility or methodological criteria]

Reichard P. Are there any glycemic thresholds for the serious microvascular diabetic complications? Journal of Diabetes and its Complications 1995;9(1):25–30. [Does not fulfil eligibility or methodological criteria]

Rushforth NB, Miller M, Bennett PH. Fasting and two-hour post-load glucose levels for the diagnosis of diabetes. The relationship between glucose levels and complications of diabetes in the Pima Indians. Diabetologia 1979;16(6):373–9. [Does not fulfil eligibility or methodological criteria]

Sabanayagam C, Liew G, Tai ES, et al. Relationship between glycated haemoglobin and microvascular complications: is there a natural cut-off point for the diagnosis of diabetes? Diabetologia 2009;52(7):1279–89. [Shared sample]

Selvin E, Ning Y, Steffes MW, et al. Glycated hemoglobin and the risk of kidney disease and retinopathy in adults with and without diabetes. Diabetes 2011;60(1):298–305. [Does not have sufficient data to reconstruct 2 × 2 table]

Sobrin L. Longitudinal validation of hemoglobin A_1c_ criteria for diabetes diagnosis: risk of retinopathy. Diabetes 2012;61(12):3074–5. [Is not a scientific article]

Sun X, Du T, Huo R, et al. Hemoglobin A1c as a marker for identifying diabetes and cardiovascular risk factors: the China Health and Nutrition Survey 2009. Acta Diabetologica 2014;51(3):353–60. [Does not fulfil eligibility or methodological criteria]

Tsugawa Y, Mukamal KJ, Davis RB, et al. Should the hemoglobin A1c diagnostic cutoff differ between blacks and whites? A cross-sectional study. Annals of Internal Medicine 2012;157(3):153–9. [Shared sample]

Vikram NK, Jialal I. Use of HbA1c in the diagnosis of diabetes and prediabetes: sensitivity versus specificity. Metabolic Syndrome and Related Disorders. 2014;12(5):255–7. [Is not a scientific article]

Woerle HJ, Pimenta WP, Meyer C, et al. Diagnostic and therapeutic implications of relationships between fasting, 2-hour postchallenge plasma glucose and hemoglobin A1c values. Archives of Internal Medicine 2004;164(15):1627–32. [Does not fulfil eligibility or methodological criteria]

Yano M, Yamakado M, Isogawa A, et al. Diagnostic sensitivity and specificity of HbAlc assay in screening of diabetes mellitus. Journal of the Japan Diabetes Society 2010;53(8):601–6. [Not available in English]
